# Increased peripheral nerve excitability and local NaV1.8 mRNA up-regulation in painful neuropathy

**DOI:** 10.1186/1744-8069-5-14

**Published:** 2009-03-25

**Authors:** Devang Kashyap Thakor, Audrey Lin, Yoshizo Matsuka, Edward M Meyer, Supanigar Ruangsri, Ichiro Nishimura, Igor Spigelman

**Affiliations:** 1 Division of Oral Biology and Medicine, School of Dentistry, University of California, Los Angeles, 10833 Le Conte Avenue Los Angeles, CA 90095-1668, USA; 2 Jane and Jerry Weintraub Center for Reconstructive Biotechnology, Division of Advanced Prosthodontics, Biomaterials, and Hospital Dentistry, School of Dentistry, University of California, Los Angeles, 10833 Le Conte Avenue, Los Angeles, CA 90095-1668, USA; 3 Neuroengineering Training Program, Biomedical Engineering Interdepartmental Program, Henry Samueli School of Engineering and Applied Science, University of California, Los Angeles, CA 90095, USA; 4 Departments of Neurosurgery and Physical Medicine & Rehabilitation, Harvard Medical School, Boston, MA, USA

## Abstract

**Background:**

Neuropathic pain caused by peripheral nerve injury is a chronic disorder that represents a significant clinical challenge because the pathological mechanisms have not been fully elucidated. Several studies have suggested the involvement of various sodium channels, including tetrodotoxin-resistant NaV1.8, in affected dorsal root ganglion (DRG) neurons. We have hypothesized that altered local expression of NaV1.8 in the peripheral axons of DRG neurons could facilitate nociceptive signal generation and propagation after neuropathic injury.

**Results:**

After unilateral sciatic nerve entrapment injury in rats, compound action potential amplitudes were increased in both myelinated and unmyelinated fibers of the ipsilateral sciatic nerve. Tetrodotoxin resistance of both fiber populations and sciatic nerve NaV1.8 immunoreactivity were also increased. Further analysis of NaV1.8 distribution revealed that immunoreactivity and mRNA levels were decreased and unaffected, respectively, in the ipsilateral L4 and L5 DRG; however sciatic nerve NaV1.8 mRNA showed nearly an 11-fold ipsilateral increase. Nav1.8 mRNA observed in the sciatic nerve was likely of axonal origin since it was not detected in non-neuronal cells cultured from nerve tissue. Absence of changes in NaV1.8 mRNA polyadenylation suggests that increased mRNA stability was not responsible for the selective peripheral mRNA increase. Furthermore, mRNA levels of NaV1.3, NaV1.5, NaV1.6, NaV1.7, and NaV1.9 were not significantly different between ipsilateral and contralateral nerves. We therefore propose that selective NaV1.8 mRNA axonal transport and local up-regulation could contribute to the hyperexcitability of peripheral nerves in some neuropathic pain states.

**Conclusion:**

Cuff entrapment injury resulted in significantly elevated axonal excitability and increased NaV1.8 immunoreactivity in rat sciatic nerves. The concomitant axonal accumulation of NaV1.8 mRNA may play a role in the pathogenesis of this model of neuropathic pain.

## Background

Neuropathic pain is a chronic disorder defined as pain initiated or caused by a primary lesion or dysfunction in the nervous system [1]; it affects 1.4% of the U.S. population and remains extremely difficult to treat, due to poorly understood etiology and a lack of well-defined molecular targets [2]. Several peripheral nerve injury-based rat models have been developed that exhibit various behavioral characteristics representative of neuropathic symptomatology, including hindpaw hypersensitivity to mechanical and thermal stimuli [3]. Aberrant hyperexcitability and ectopic burst discharge of primary sensory neurons in these models is widely considered to be the major contributor to neuropathic pain symptomatology. Functional studies have implicated ion channels, including sodium channels, in this pathology. In particular, loss-of-function studies using antisense oligodeoxynucleotides and siRNA have highlighted the importance of the NaV1.8 tetrodotoxin resistant (TTX-r) sodium channel in the neurophysiological and behavioral effects observed in spinal nerve ligation (SNL) and chronic constriction injury (CCI) models of peripheral neuropathy [4-7]. Among TTX-r sodium channels, Nav1.8 has been shown to contribute substantially to action potential electrogenesis in dissociated DRG neurons [8,9] and is expressed in both C- and A-fiber populations [10,11]. NaV1.8 has a higher inactivation threshold, slower inactivation kinetics, and faster recovery from inactivation than TTX-sensitive Na^+ ^channels [12,13], suggesting that its local upregulation could facilitate signaling during neuropathic pain. Additionally, it has been found that NaV1.8-null transgenic mice do not exhibit abnormal neurophysiology in injured neuroma tissue [14].

These and other studies have suggested that peripheral NaV1.8 increases in sensory neuron axons contribute to peripheral hypersensitivity. For example, in human patients with various neuropathies such as causalgia and brachial plexus injury, NaV1.8 has been shown to accumulate in peripheral axons near the injury site [15-17] and in a causalgic finger [18]. Increases in NaV1.8-like immunoreactivity in axons of the rat sciatic nerve were demonstrated after CCI [11]. Also, after SNL injury sciatic nerve NaV1.8-like immunoreactivity increased selectively in 'uninjured' axons; this was accompanied by increased TTX-resistance of the C-fiber compound action potential, suggesting a functional peripheral upregulation of NaV1.8 [5]. However, discrepancies between these findings and the observed reduction of NaV1.8 protein and mRNA expression in the cell bodies of injured sensory neurons [19], as well as the normal development of neuropathic pain behavior in NaV1.8–null mice [20,21] continue to raise the question of what role, if any, is played by NaV1.8 in the development of neuropathic pain. Also, the mechanism by which peripheral translocation of NaV1.8 occurs in the CCI model is unclear [11].

To address the mechanisms of NaV1.8 translocation and its potential relationship to hyperexcitability, we studied sciatic nerve excitability and NaV1.8 expression in a rat model of painful neuropathy induced by sciatic nerve entrapment (SNE). SNE is a variation on CCI in which the loose ligatures placed around the sciatic nerve are replaced by chemically inert fixed-diameter polyethylene cuffs, resulting in decreased variability across animals [22]. We found increased sciatic nerve axonal excitability and TTX-resistance proximal to the injury. This was concommitant with increased NaV1.8 immunoreactivity in proximal axons and decreased immunoreactivity in the cell bodies of DRG neurons on the side of the injury, analogous to previous findings after CCI [11]. Importantly, we observed selective and large increases in sciatic nerve NaV1.8 mRNA in the absence of changes in NaV1.8 mRNA within DRG or changes in NaV1.8 polyadenylation. Our data collectively introduce the possibility that NaV1.8 mRNA could be peripherally transported from the neuronal cell bodies to the sciatic nerve and locally translated, thereby contributing to axonal hyperexcitability and neuropathy symptoms.

## Results

### Sciatic nerve excitability and TTX-resistance after SNE

**Figure 1:**
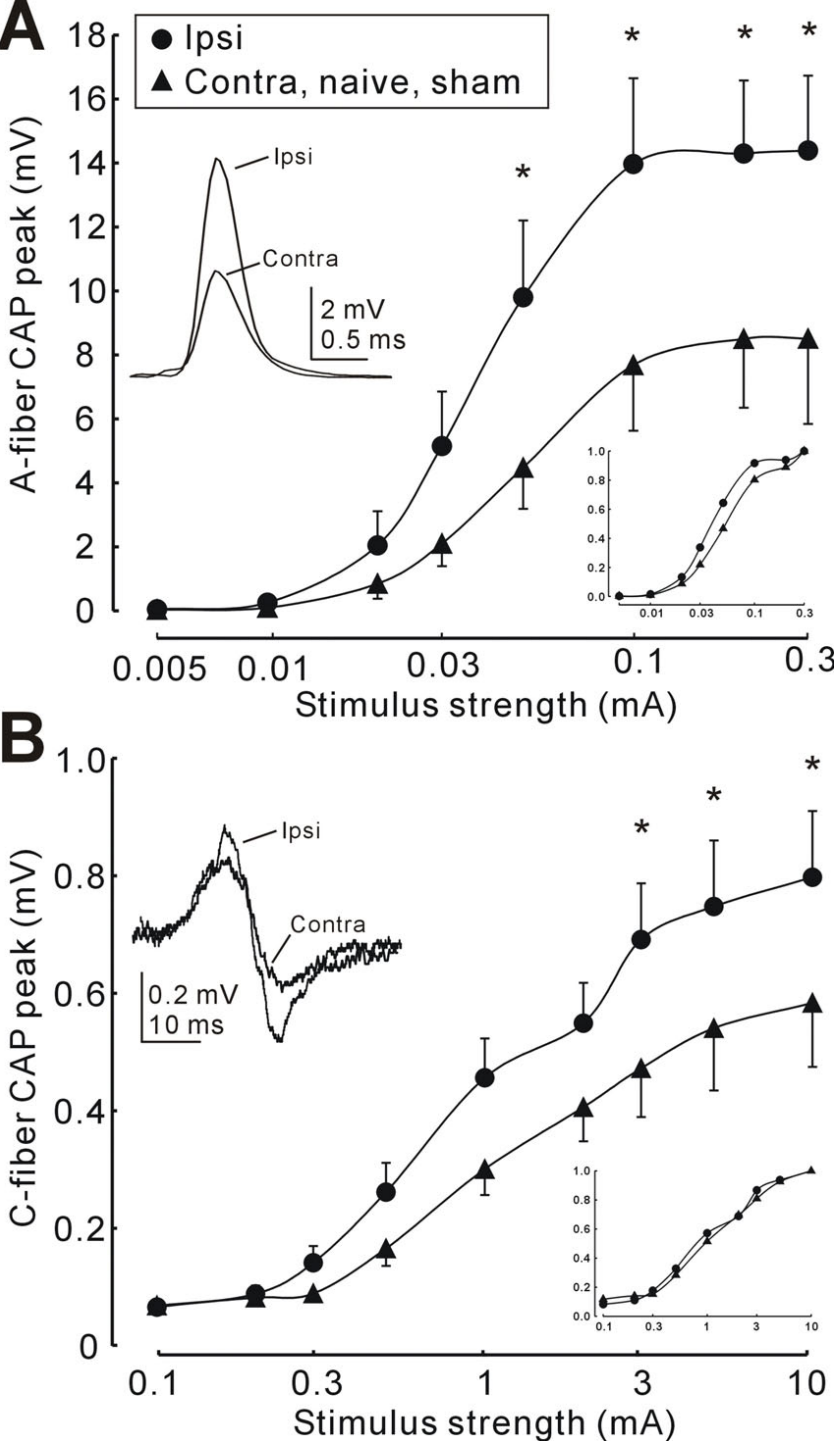
**Increased excitability of ipsilateral sciatic nerve after SNE**. A: Stimulus-output plot of A-fiber CAP peak for sciatic nerve ipsilateral to SNE (n = 8) compared to preparations from contralateral, naïve or sham control rats (n = 8). Inset (left) shows examples of ipsilateral and contralateral A-fiber CAP with peaks aligned for clarity (0.1 mA stimulus). *, p < 0.05 (two-way ANOVA, Tukey post-hoc). Inset (bottom right) shows normalized data with error bars removed for clarity. Note the leftward shift of data from ipsilateral nerves. B: Stimulus-output plot of C-fiber CAP peak for sciatic nerve ipsilateral to SNE compared to preparations from contralateral, naïve or control rats. Inset (left) shows examples of ipsilateral and contralateral C-fiber CAP (10 mA stimulus). Inset (bottom right) shows normalized data with error bars removed for clarity. Stimulus durations were 0.1 ms and 0.5 ms for A- and C-fiber CAP, respectively throughout. Tissue was harvested 2 weeks after SNE or sham surgery.

The CAP response amplitudes from contralateral, naïve control nerves, and ipsilateral sham nerves were similar and therefore grouped together. 2 weeks after SNE, the amplitudes of both A- and C-fiber CAPs were significantly increased in ipsilateral sciatic nerves when compared to control, sham or contralateral nerves (Fig. 1). The increases were observed in the plateau responses to maximal stimulation (Fig. 1A, B) Although leftward shifts in the stimulus-response relationsips were also detected for the A-fiber and to a lesser extent the C-fiber CAP after normalizing responses to maximal amplitudes, these shifts were not statistically significant (Fig. 1A and 1B insets). The conduction velocity (CV) of the peak A-fiber CAP was not different between nerves ipsilateral to SNE (32.4 ± 1.5 m/s) and control, sham or contralateral nerves (29.9 ± 1.7 m/s). The CV of the ipsilateral C-fiber CAP 1.2 ± 0.1 m/s) was also similar to that of control, sham and contralateral nerves (1.1 ± 0.1 m/s).

**Figure 2:**
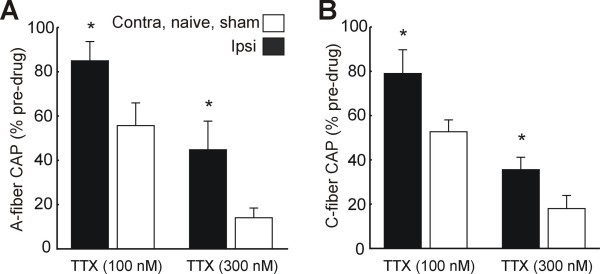
**Increased TTX-resistance of the sciatic nerve after SNE**. A: TTX-resistance of the A-fiber sciatic nerve compound action potential (CAP) ipsilateral to SNE (n = 8) compared to contralateral, naïve and sham surgery nerves (n = 8). CAP response is expressed as a % of recorded amplitude before TTX administration. A-fiber CAP stimulus was 0.1 mA, 0.1 ms throughout. B: Changes in TTX-resistance of the C-fiber sciatic nerve CAP after SNE. C-fiber CAP stimulus was 10 mA, 0.5 ms throughout. * indicates significant difference from control nerve sensitivity to TTX (p < 0.05, two-way ANOVA, Tukey post-hoc). Tissue was harvested 2 weeks after SNE or sham surgery.

Concentration-dependent decreases in CAP amplitude were detected within 30 sec after TTX (0.1–1 μM) was applied in the recording chamber (p < 0.05, two-way ANOVA with Tukey post-hoc) and measurements were obtained after 20–25 min to ensure a full drug response. The C-and A-fiber CAPs in both injured and uninjured nerves were blocked completely in the presence of 1 μM TTX (not shown). At lower concentrations, the TTX-resistance of both the A-fiber and C-fiber CAPs was significantly increased in nerves ipsilateral to SNE (p < 0.05, two-way ANOVA with Tukey post-hoc) (Fig. 2A, B).

### Peripheral nerve NaV1.8 immunoreactivity after SNE

**Figure 3:**
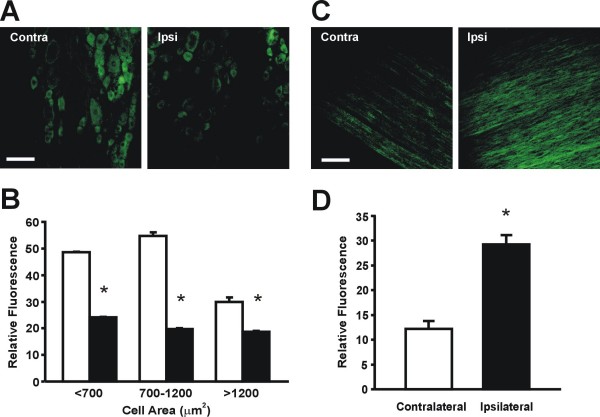
**NaV1.8 protein is down-regulated in the DRG and up-regulated in the nerve after SNE**. A: Nav1.8-ir in representative DRG sections after SNE. B: Quantification of fluorescence intensity in DRG neurons. Neurons are classified by cell area. □: contralateral. ▪: ipsilateral. DRG measurements were from 3 contralateral and 6 ipsilateral sections (n = 3. *; p < 0.05, one-way ANOVA, Tukey post-hoc). **C: **NaV1.8-ir in representative sections of sciatic nerve after SNE. D: Quantification of fluorescence intensity in sciatic nerve. Measurements were made from 8 contralateral and 8 ipsilateral sections. (n = 3, *; p < 0.05, t-test). Scale bar: 100 μm for A and C. Tissue was harvested 2 weeks after SNE. For both DRG and nerve sections, average intensity was measured using ImageJ software. Output intensity values were on a 0–255 scale based on the 255 shades of grey present in the image, with 0 representing black and 255 representing white. The output numerical intensity value was defined as "relative fluorescence."

At two weeks after SNE injury, NaV1.8-like immunoreactivity in the ipsilateral L4 and L5 dorsal root ganglia (DRG) was reduced (Fig. 3A, B). Specifically, the staining intensities of NaV1.8+ small-, medium-, and large diameter neurons were all significantly decreased (p < 0.05, one-way ANOVA with Tukey post-hoc; Fig. 3B). In marked contrast to the DRG, NaV1.8-like immunoreactivity increased more than two-fold in ipsilateral nerves (p < 0.05, t-test; Fig. 3C, D).

### Detection of NaV1.8 mRNA in sciatic nerves

**Figure 4:**
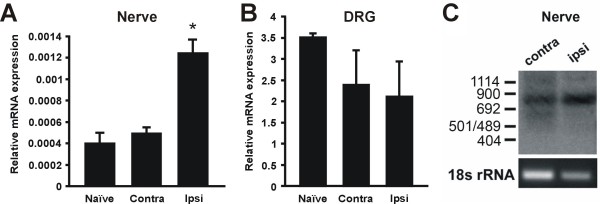
**Detection of NaV1.8 mRNA in the sciatic nerve**. A. NaV1.8 mRNA levels were significantly increased in the sciatic nerve ipsilateral to the injury site as compared to contralateral and uninjured DRG. (n = 3; p < 0.05, one-way ANOVA with Tukey post-hoc). B. NaV1.8 mRNA levels in SNE-injured DRG were not significantly different from those of uninjured DRG (n = 4 for uninjured DRG and n = 3 for all other samples). C: Southern blot of the Nav1.8 3' RACE product from contralateral and ipsilateral sciatic nerve. Note the presence of a distinct band representing the expected 3'RACE product of 830 bp. Sequencing of the RACE product confirmed the presence of the 3' end of the NaV1.8 coding region as well as the NaV1.8 3' un-translated region and polyA tail. Tissue was harvested 2 weeks after SNE.

Real-time PCR detected the presence of NaV1.8 mRNA in the sciatic nerve and revealed a significant up-regulation in the ipsilateral nerve as compared to the contralateral and uninjured nerve (p < 0.05, one-way ANOVA with Tukey post-hoc; Fig. 4A). In contrast, ipsilateral, contralateral, and uninjured DRG showed no significant differences in NaV1.8 mRNA levels (Fig. 4B). To confirm that the NaV1.8 PCR signals represent mRNA and not genomic DNA contamination, 3'RACE PCR was performed. Southern blot hybridization analysis showed a positive band at the predicted size of 830 bp in both contralateral and ipsilateral sciatic nerve samples (Fig. 4C). After agarose gel separation and cloning of amplicons, DNA sequencing positively identified the 3' end of the coding region and the 3' untranslated region of rat Nav1.8 as well as an additional poly A tail (data not shown).

### Evidence for neuronal origin of NaV1.8 mRNA in sciatic nerve

**Figure 5:**
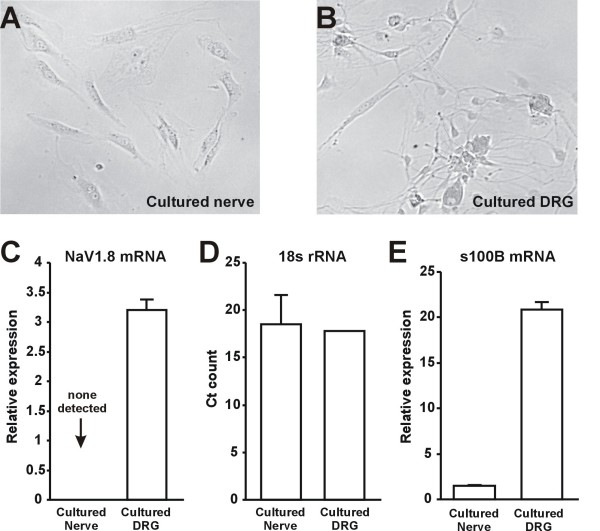
**Axonal origin of sciatic nerve NaV1.8 mRNA**. A, B: Representative images of cells cultured from the sciatic nerve and dorsal root ganglia. Cells with Schwann- and fibroblast-like morphology were present in both nerve and DRG cultures, whereas neurons were only present in the DRG culture. C: Expression of 18s rRNA in nerve and DRG cultures (n = 3). Expression is given in terms of the absolute cycle count where signal was first detected. D. Expression of NaV1.8 mRNA in nerve and DRG cultures (n = 3). Relative expression was normalized externally against the DRG sample with the lowest NaV1.8 expression and internally against 18s expression rRNA expression. E. Expression of s100B mRNA in nerve and DRG cultures (n = 3). Relative expression was normalized externally against the nerve sample with the lowest s100B expression and internally against 18s expression rRNA expression. Strong expression was observed in both cultures with Ct values of 26–30. However, relative expression was still higher in DRG cultures.

We hypothesized that if sciatic nerve NaV1.8 mRNA was of non-neuronal origin, it would be detectable in cultures of dissociated sciatic nerves where there are no neuronal somata and neuronal axons have degenerated. No real-time PCR signal was detected for NaV1.8 in sciatic nerve cultures (Fig 5A, C), whereas robust NaV1.8 expression was observed in DRG cultures that served as a positive control (Fig. 5B, C). However, similar signals for 18s rRNA were detected in the sciatic nerve and DRG cultures (Fig. 5D), suggesting similar concentrations of template cDNA. Additionally, we were able to detect strong expression of s100B, a Schwann cell marker, in both sciatic nerve and DRG cultures, with Ct values ranging from 26–30. Interestingly, although s100B expression was strong in nerve cultures, it was still stronger in DRG cultures (Fig. 5E).

### Selectivity of peripheral nerve NaV1.8 mRNA up-regulation after SNE

**Figure 6:**
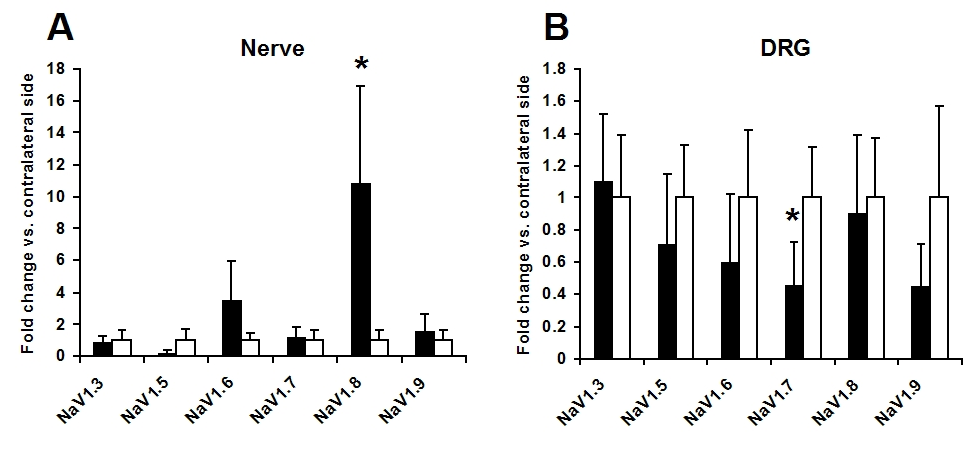
**Selective peripheral translocation of NaV1.8 mRNA after SNE injury**. A: Sodium channel mRNA levels in naive, contralateral, and ipsilateral sciatic nerve. Black columns: ipsilateral side. White columns: contralateral side. B: Sodium channel mRNA levels in contralateral and ipsilateral L4 and L5 DRG. Black columns: ipsilateral side. Grey columns: contralateral side. Note that NaV1.8 showed a significant 10.8-fold increase in the ipsilateral sciatic nerve (*; p < 0.05, Mann-Whitney) and that this increase did not occur in the DRG. NaV1.6 showed a 3.48-fold increase in the nerve, but this did not reach statistical significance (p = 0.19, Mann-Whitney). NaV1.5 showed an 80.2% decrease in the ipsilateral nerve, but this also did not reach statistical significance (p = 0.079, Mann-Whitney) Ipsilateral NaV1.5, NaV1.6, NaV1.7, and NaV1.9 mRNA levels decreased by 28.6%, 39.8%, 54.1%, and 54.9%, respectively, but the downregulation only reached statistical significance for NaV1.7 (p < 0.05, Mann-Whitney). All samples were normalized externally against the mean contralateral expression and internally against 18s rRNA expression (n = 3–7). Means and standard errors are shown for convenience; parametric analyses of non-parametric data returned similar results.

After confirmation of the presence of NaV1.8 mRNA in the sciatic nerve, we compared steady-state levels of mRNA for NaV1.3, NaV1.5, NaV1.6, NaV1.7, NaV1.8, and NaV1.9 between ipsilateral and contralateral segments of the sciatic nerve immediately proximal to the injury site (Fig. 6A). Ipsilateral NaV1.8 mRNA was significantly up-regulated by 10.8-fold (p < 0.05, Mann-Whitney). Ipsilateral NaV1.6 mRNA expression was increased by 3.48-fold, but this difference did not reach statistical significance (p > 0.05, Mann-Whitney). None of the other sodium channels showed significant up-regulation in the sciatic nerve (p > 0.05, Mann-Whitney). We further examined mRNA expression of the same sodium channels in the DRG at the same time point. Ipsilateral NaV1.5, NaV1.6, NaV1.7, and NaV1.9 mRNA levels decreased by 28.6%, 39.8%, 54.1%, and 54.9%, respectively, however only the decrease was only statistically significant for NaV1.7 (p < 0.05, Mann-Whitney; Fig. 6B).

### Polyadenylation of peripheral NaV1.8 mRNA

**Figure 7:**
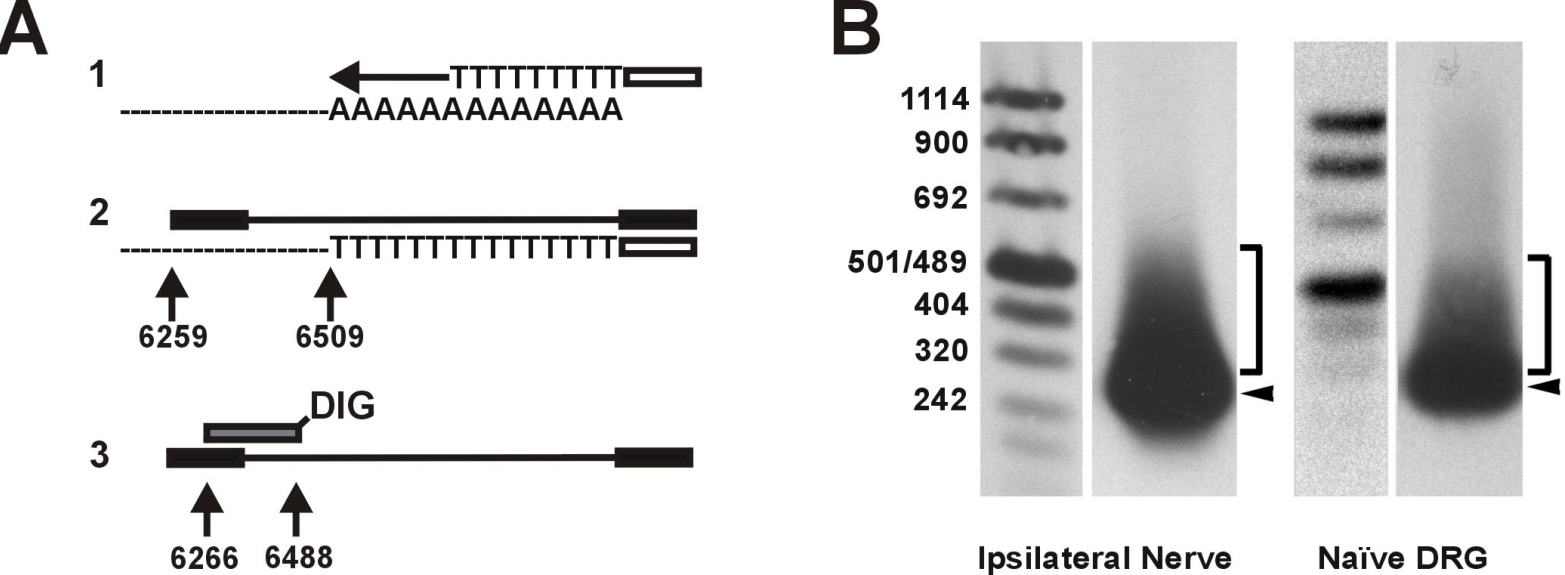
**NaV1.8 mRNA polyadenylation does not increase in the injured sciatic nerve**. A: Schematic representation of PolyA Tail (PAT) assay. 1. Reverse transcription with oligo dT-Adaptor primer. 2. NaV1.8-specific PCR amplification with Adaptor primer. Amplicon length depends on polyA tail length. 3. Southern blot detection with digoxygenin (DIG)-labeled NaV1.8-specific probe after gel electrophoresis. Multiple polyA tail lengths result in smearing and multiple bands. B: Southern blot of PAT amplicons. ◂: NaV1.8 mRNA from both ipsilateral nerve and uninjured DRG tissue predominantly exhibits a discrete polyA tail < 50 bases long, with additional polyadenylation occurring out to 200 bases. Tissue was harvested 2 weeks after SNE.

To investigate potential inhibition of peripheral NaV1.8 mRNA degradation by polyadenylation, we conducted the polyA tail length assay as originally described by Salles and Strickland [23]. NaV1.8 mRNA isolated from both injured nerve and uninjured DRG primarily exhibited a discrete polyA tail of about 50 bases, with additional polyadenylation occurring out to 200 bases (Fig. 7). However, there were no apparent differences in the maximum polyA tail length or smearing pattern.

## Discussion

The NaV1.3, NaV1.8, and NaV1.9 sodium channels are differentially regulated after nerve injury [24], and the TTX-resistant NaV1.8 and NaV1.9 are selectively distributed in peripheral sensory neurons [24]; these properties have identified them as potential molecular targets for neuropathic pain. Antisense oligodeoxynucleotide and siRNA studies suggest that sensory neuron NaV1.8 expression and spinal cord NaV1.3 expression are important for behavioral phenotypes and aberrant neurophysiology in rats with peripheral nerve injuries [4-7,25]. Rat loss-of-function studies have not yet been performed for NaV1.9, but studies of NaV1.9-null transgenic mice suggest involvement in inflammatory rather than neuropathic pain [26,27].

The requirement of NaV1.8 and NaV1.3 for neuropathic pain remains controversial, however, as neuropathic pain behavior develops normally in NaV1.8- and NaV1.3-null mice [20,21,28], as well as in mice treated with diptheria toxin to selectively destroy all postmitotic sensory neurons expressing NaV1.8 [29]. It is possible that the discrepancy between these findings and those of rat loss-of-function studies could arise from compensation for NaV1.8 or NaV1.3 deletion, as well as species differences, although it has also been shown that intrathecal antisense knockdown of NaV1.3 did not reverse pain symptoms in a rat spared nerve injury (SNI) model [30].

We hypothesized that SNE injury would produce sensory neuron hyperexcitability which would be related to altered local expression of NaV1.8. We found that SNE injury in rats induced a unique electrophysiological phenotype characterized by increased sciatic nerve CAP amplitude in both C- and A-fiber populations (Fig. 1). The C-fiber increase likely contributed to the thermal hypersensitivity we have previously observed in this model, while Aδ-fiber excitability would be expected to contribute to the observed mechanical hypersensitivity [31]. However, the overall magnitude of the A-fiber CAP increase and lack of change in conduction velocity suggest increased excitability of Aβ-fibers instead of or in addition to Aδ-fibers. Aβ-fibers are not normally involved in nociception [32] with the exception of a small subpopulation [33], but phenotypic changes in Aβ-fibers have been reported after nerve injuries including SNE [34,35] that result in nociceptive afterdischarge in spinal wide dynamic range neurons [35]. It is possible that such a phenotypic switch might have been involved in the observed A-fiber CAP enhancement and concomitant mechanical or thermal hypersensitivity.

It is notable that increased A- and C-fiber CAP amplitudes persisted even after the stimulus-output plot reached a plateau (Fig. 1). Because increased CAP amplitude is typically interpreted as a recruitment of more axons, a stimulus-output plateau suggests that all available axons have been recruited. A further CAP increase could potentially arise from a change in the total number of axons; however, after SNE the numbers of large- and small-diameter afferents do not change proximal to the injury site, where the recorded tissues were sampled [22]. It is therefore more likely that the observed CAP increase represents peripherally increased Na^+ ^conductance in individual axons. It is further likely that such an increase in conductance would be at least partially mediated by TTX-resistant channels, as CAP TTX resistance increased concomitantly with CAP amplitude for both C- and A-fibers after SNE injury (Fig. 2). An alternative explanation for these observations is that increased expression of TTX-sensitive Na^+ ^channels could increase both the CAP amplitudes and the safety factor for axonal action potential conduction during TTX application. Another possibility we considered was the upregulation of a third TTX-R channel, Nav1.5, which is normally expressed in sensory neurons during development but declines to very low levels at birth and adulthood [36]. Nav1.5 exhibits faster kinetics than Nav1.8 and is significantly more sensitive to TTX (IC_50 _~2 μM) than Nav1.8 or Nav1.9 [36]; thus upregulation of Nav1.5 could potentially account for the observed increases in TTX-resistance, including the blockade of A and C-fiber CAP conduction by 1 μM TTX after SNE. However, we did not observe increased NaV1.5 mRNA expression in the DRG or the sciatic nerve after SNE.

Much larger increases in CAP TTX-resistance, and only in C-fibers (resistant to block by 100 μM TTX), were reported for the SNL model [5], underscoring major differences between the SNE and SNL models. SNL is thought to cause extensive Wallerian degeneration of injured axons from L5 and L6 DRG, leaving only uninjured L4 axons in the sciatic nerve [5]. In contrast, CCI and SNE cause selective degeneration of Aβ-fibers distal to the constriction site with some remyelination occurring 2–4 weeks after injury [37,38]; this partial, distal degeneration leaves proximal injured and uninjured axons structurally intact.

We also found that sciatic nerve NaV1.8 immunoreactivity was increased after SNE injury, while immunoreactivity within cell bodies of DRG sensory neurons ipsilateral to SNE was reduced. Similar findings were previously reported for the CCI model [11], underscoring simmilarities with SNE. This peripheral translocation of Nav1.8 together with increased TTX-resistance suggests involvement of peripherally accumulated NaV1.8 in sciatic nerve hyperexcitability after SNE injury.

Several mechanisms may underly the peripheral translocation of NaV1.8 protein observed in multiple injury models. In the present study, we detected ipsilaterally decreased DRG NaV1.8 immunoreactivity (Fig. 3), but NaV1.8 mRNA levels in the DRG were not affected by SNE injury (Figs. 4 &5). Similar post-CCI immunohistochemical redistribution of NaV1.8 to sciatic nerve axons without increased DRG mRNA expression previously led Novakovic et al. to suggest peripheral trafficking of existing NaV1.8 protein with increased local insertion [11]. Decreased NaV1.8 mRNA levels and attenuated NaV1.8 currents that could be compatible with such a mechanism were also observed in injured small-diameter DRG somata cultured from CCI-injured rats [39]; NaV1.8 protein levels in injured neurons within the DRG were also shown to decrease in rats after SNL, CCI, SNI, and axotomy injuries [24]. However, while the majority of *de novo *protein synthesis is believed to occur in DRG somata, an increasing number of studies suggest that axons contain mRNAs such as β-actin, cofilin and RhoA that are locally translated in response to guidance cues during nervous system development [40-42]. Recently, intra-axonal CREB mRNA translation was shown to be essential for neuronal survival in response to nerve growth factor application [43]. We thus hypothesized that axonal NaV1.8 mRNA accumulation might contribute to the increased immunoreactivity in the nerve proximal to SNE injury.

To address this hypothesis, we first determined the presence of NaV1.8 mRNA in the sciatic nerve using RT-PCR (Fig. 4 &5; also see [44]. The 3'RACE sequencing identified a polyA tail in NaV1.8 PCR amplicons, suggesting that our PCR data did not reflect genomic DNA contamination. In addition, we determined the cellular origin of NaV1.8 transcripts because sciatic nerve contains non-neuronal cells such as Schwann cells and fibroblasts in addition to neuronal axons. Cells cultured from both naïve DRG and sciatic nerve showed expression of s100B, confirming the presence of Schwann and/or satellite 'glial' cells. However, NaV1.8 expression was not detected in the nerve cultures (Fig. 6), suggesting that NaV1.8 transcripts detected in intact nerve tissue were likely derived from axons.

Significant up-regulation of NaV1.8 mRNA was demonstrated in the ipsilateral sciatic nerve when compared to the contralateral or uninjured nerve (Fig. 4). To explore the mechanism of the peripheral NaV1.8 mRNA increase, we examined the polyadenylation state. There were no differences in maximum NaV1.8 polyA tail length or smearing pattern between injured nerve and uninjured DRG samples (Fig. 7). Therefore, intra-axonal polyadenylation resulting in reduced turnover and increased steady-state mRNA levels [45] was ruled out as a mechanism of increased NaV1.8 mRNA accumulation in SNE axons.

Strikingly, simultaneous analysis of multiple sodium channels further demonstrated that after SNE injury, steady-state NaV1.8 mRNA was significantly increased in the ipsilateral nerve by nearly 11-fold when compared to the contralateral nerve. In contrast, we were unable to observe any changes in ipsilateral mRNA levels of NaV1.3, NaV1.6, NaV1.7, or NaV1.9 (Fig. 5). It is unlikely that the selective NaV1.8 mRNA increase was the indirect result of ultrastructural changes, as both the fiber number and composition remain unaltered at the sampling location proximal to the injury site [37]. These findings thus suggest a novel mechanism of selective NaV1.8 mRNA accumulation in injured axons.

A recent study of rat saphenous nerve neuromas revealed increased local translation of structural and metabolic proteins, although no changes were observed in the steady-state protein levels of NaV1.8, NaV1.3, or the α 2δ calcium channel [46]. It has also been shown that axonally transported importin β and vanilloid receptor mRNAs can be locally translated in sciatic nerve axons after nerve injury and dorsal horn synaptic terminals during inflammation, repsectively [47,48]. Various mRNAs coding cytoskeletal and metabolic proteins have been found to be axonally transported and locally translated in a cultured axonal preparation [49]. Thus, we propose here that enhanced and selective peripheral transport is the most likely mechanism for axonal NaV1.8 mRNA up-regulation, and that local mRNA accumulation could contribute to the observed increase in sciatic nerve NaV1.8 immunoreactivity. Our findings also support the involvement of peripheral NaV1.8 protein in sciatic nerve hyperexcitability after neuropathic injury.

## Conclusion

While our experimental design did not permit the direct discrimination of somatically- and axonally-derived protein, our findings collectively introduce the possibility that sodium channel mRNA could be peripherally transported from the DRG to the sciatic nerve and locally translated. If this is the case, the local up-regulation of NaV1.8 mRNA shown here after injury may at least partially contribute to the observed peripheral increases in NaV1.8 protein levels and nerve excitability, which in turn may play a role in the aberrant nociception that characterizes neuropathic pain.

## Methods

### Sciatic nerve entrapment (SNE)

Surgery was performed as described previously [22] using adult male Sprague-Dawley rats weighing 200–250 g. Briefly, three Tygon^® ^cuffs (length = 1 mm, outer diameter = 2.28 mm, inner diameter = 0.76 mm) were placed around the exposed sciatic nerve proximal to the trifurcation.

### Immunohistochemistry

At 2 weeks after injury, rats were anesthetized with pentobarbital (100 mg/kg, i.p.) and fixed by intracardiac perfusion of 500 ml ice-cold 4% paraformaldehyde and 0.14% picric acid in 0.1 M phosphate buffer (PB), pH 7.4. Ipsilateral and contralateral L4 and L5 DRG were harvested along with sciatic nerve segments. Tissue was post-fixed for 24 hrs and cryoprotected overnight in 30% sucrose in 0.1 M PB. Samples were mounted in Tissue-Tek (Sakura Finetek USA, Inc., Torrance, CA), and cryosectioned at 25 μm (DRG) or 20 μm (sciatic nerve). Free-floating sections were incubated overnight with an anti-NaV1.8 antibody (1:2000 dilution, in PBS containing 5% NGS and 0.3% Triton X-100). The NaV1.8 antibody, which was previously extensively characterized, ^15 ^was generously provided by Dr. John Wood, London, UK. Sections were then incubated in a FITC-labeled goat anti-rabbit secondary antibody (Molecular Probes, Carlsbad, CA) at 1:500 dilution. The sections were mounted in an anti-fade medium (ProLong, Molecular Probes, Carlsbad, CA). To minimize variability between specimens, ipsilateral and contralateral tissues were processed simultaneously and representative sections grouped on the same slides.

### Confocal Microscopy

Confocal fluorescence images were taken at 20× magnification on a Leica TCS-SP confocal fixed-stage upright microscope (Heidelberg, Germany) equipped with argon (488 nm excitation: JDS Uniphase) and DPSS diode (561 nm excitation: Melles-Griot) lasers. All laser excitation and detection parameters were kept constant across all samples. Image stacks were averaged and given identical high and low intensity thresholds using LCS lite software (Leica, Heidelberg, Germany). For DRG images, neuronal area was measured by outlining NaV1.8 immunoreactive somata and then calculating the area with ImageJ software . Fluorescence intensity was measured by outlining the cytosolic region of NaV1.8 immunoreactive somata; outlines were in the shape of an annulus, to exclude the darker nuclei. Average cytosolic fluorescence intensity was then measured using ImageJ software. When overlapping NaV1.8-immunoreactive somata were detected, the overlapping regions were excluded from intensity analysis. For sciatic nerve analyses, 1–2 111 × 320 pixel rectangles were drawn on each image such they did not cover any gap, break, or artifact. The average intensity within each rectangle was measured using ImageJ software. For both DRG and nerve sections, output intensity values were on a 0–255 scale based on the 255 shades of grey present in the image, with 0 representing black and 255 representing white. The output numerical intensity value was defined as "relative fluorescence."

### Surgery, tissue collection and preparation for electrophysiology

At 2 weeks after injury, rats were anesthetized with sodium pentobarbital (50 mg/kg) and L4 and L5 DRG were excised with their dorsal roots, the ventral roots, the spinal nerves and a length of attached sciatic nerves cut just proximal to the site of entrapment. The preparations were further trimmed at 0–4°C in the low-Na^+ ^artificial cerebrospinal fluid (ACSF) composed of (in mM): NaCl, 62; KCl, 3.5; NaH_2_PO_4_, 1.25; CaCl_2_, 2; MgCl_2_, 2; NaHCO_3_, 26; glucose, 10 and sucrose, 124. Isolated DRG and sciatic nerve sections were then placed in cryogenic vials, frozen in liquid nitrogen and stered at -80°C. Alternatively, sections of sciatic nerve were then transferred to normal ACSF (20–23°C) composed of (in mM): NaCl, 124; KCl, 3.5; NaH_2_PO_4_, 1.25; CaCl_2_, 2; MgCl_2_, 2; NaHCO_3_, 26 and glucose, 10. The ACSF was continuously bubbled with a 95/5% mixture of O_2_/CO_2_. For recording, preparations were transferred to a custom recording chamber (0.3 ml volume) that was perfused (2.7 ml/min) with oxygenated ACSF at 34.5°C. TTX (0.1–1 μM, Sigma) was dissolved and applied in ACSF.

### Compound action potential recording

Compound action potentials (CAPs) were recorded from the sciatic nerves using a suction recording electrode [50,51]. Signals from the recording electrode and the artifact suppression electrode were fed into a differential amplifier (Dam 50, WPI, Sarasota, FL) in AC mode. A calibration pulse (± 2 V across a 100 MΩ series resistor) was always included in the CAP recordings to estimate the resistance at the nerve/recording electrode junction [50,51]. Signals from CAP recordings were further amplified (Brownlee Precision Instruments, model 440, Santa Clara, CA). Amplified signals were digitized at 10–20 kHz via the Digidata 1200B interface (Molecular Devices, Union City, CA) and displayed on a computer screen using the pCLAMP 8 software package (Molecular Devices). Data were analyzed offline using the pCLAMP 8 software package (Molecular Devices). Since CAP amplitude/area varies linearly with changes in this resistance [50,51], the CAP signal was adjusted based on the calibration pulse amplitude changes during analysis of any given recording. All data are presented as mean ± standard error of the mean (SEM).

### Cell Culture

Sciatic nerve sections and lumbar DRG were extracted as above and cut into small pieces in ice-cold Hank's balanced salt solution (HBSS) with 20% fetal bovine serum (FBS). The tissue was incubated for 75 min at 37°C in HBSS with 0.125% collagenase P (Roche, Indianapolis, IN), and 0.05% DNAse. 0.25% trypsin was added for 5 min, after which the tissue was centrifuged for 5 min at 2000 rpm. After washing with 20% FBS in HBSS, the cell pellet was resuspended in HBSS containing 0.295% MgSO_4 _and 0.05% DNAse. The tissue was then triturated 10 times with a fire-polished siliconized Pasteur pipette and centrifuged for 5 min at 2000 rpm. The resulting cell pellet was resuspended in 1 ml of F12 nutrient mixture with 10% FBS, and 300 μl were plated into each of 3 wells of a 12-well cell culture plate. The plate was incubated at 37°C for 1 hr, and then another 700 μl of medium was added. The medium was then changed every 2 days. After 4 days, culture plates were washed in PBS, snap-frozen in liquid nitrogen, and then stored at -80°C. For RNA extraction, culture plates were thawed, and 350 μl of buffer RLT from the RNEasy Plus Mini Kit (Qiagen, Valencia, CA) were immediately added to each well. Wells were incubated for 15 minutes at room temperatue and scraped with pipette tips. The buffer RLT containing scraped cells from each well was then placed into separate RNAse-free 1.5 mL microcentrifuge tubes. The RNEasy Plus protocol with gDNA eliminator columns was then followed for RNA extraction.

### Quantitative real-time reverse-transcription polymerase chain reaction

At 2 weeks after injury, rats were anesthetized (50 mg/kg pentobarbital, i.p.), and ipsilateral L4 and L5 DRG were harvested together with sciatic nerve segments. RNA was extracted using a combination of phenol/chloroform extraction and glass fiber purification (Trizol, Invitrogen, Carlsbad, CA, and RNAqueous, Ambion, Austin, TX), DNAse treated (Ambion, Austin, TX), and reverse-transcribed (Superscript III, Invitrogen, Carlsbad, CA) using random hexamer primers. Samples were subjected in triplicate to real-time PCR under default cDNA conditions (Applied Biosystems, Foster City, CA) using a ready-made 18s rRNA primer and probe set (Applied Biosystems, Foster City, CA) as an internal control. Ready-made Taqman expression assay sets (Applied Biosystems, Foster City, CA) were used for PCR amplification of various sodium channel cDNAs; the expression assay IDs were: NaV1.3, Rn00565438_m1; NaV1.5, Rn00565502_m1; NaV.16, Rn00570506_m1; NaV1.7, Rn00581647_m1; NaV1.8, Rn_00568393; NaV1.9, Rn00570487_m1.

### 3'Rapid amplification of cDNA ends (3'RACE)

At 2 weeks after injury, total RNA (800 ng) extracted separately from ipsilateral and contralateral sciatic nerve was converted to first strand cDNA and then subjected to 3'RACE PCR using the SMART RACE cDNA Amplification Kit (Clontech, Mountain View, CA). The anchoring primer for the 3'RACE PCR reaction was 5'-GTTTATGGCGACAATCTCTCCAAAG-3' (Tm = 65°C), located approximately 830 bp upstream of the NaV1.8 mRNA polyA tail. Approximately 18 ng of first strand cDNA template was consistently used for all samples. Samples were denatured at 94°C for 30 sec, annealed at 68°C for 30 sec, and elongated at 72°C for 3 min. First strand cDNA was amplified for 35 cycles with the anchoring primer and oligo d(T) primer. Southern blot hybridization analysis was used to identify positive PCR amplicons containing NaV1.8 3'end sequence. The DIG-labeled DNA probe against the NaV1.8 3' end coding sequence was synthesized using the PCR DIG Probe Synthesis Kit (Roche, Indianapolis, IN) with forward primer 5'-GGAAGACCTCTCAGCCACAG-3' and reverse primer 5'-CAGGGTGTTGGAGAGTGTCA-3'. After hybridization, the membrane was washed, blocked, incubated with anti-DIG antibody, and detected according to the manufacturer's instructions. Chemiluminescence was detected by a LAS-3000 Luminescent Image Analyzer (Fujifilm, Tokyo, Japan). 18S ribosomal RNA was amplified from the contralateral and ipsilateral sciatic nerve RNA samples for use as an internal control during PCR.

### PolyA tail length assay

At 2 weeks after injury, total RNA was extracted as described above. Reverse transcription was performed using 5'-GCGAGCTCCGCGGCCGCGTTTTTTTTTTTT-3' as the primer. Reaction mixtures were prepared on a block heated to 94°C and then subjected to 35 cycles of PCR amplification using high fidelity Ex Taq polymerase (Takara, Otsu, Japan). Each cycle consisted of 30 sec denaturation, 1 min annealing, and 2 min extension. The primers were 5'-ACTCCTCCATTCTGACGTCCCTTCC-3' and 5'-GCGAGCTCCGCGGCCGCGTTTTTTTTTTTT-3,' and the annealing temperature was 68°C.

Digoxigenin-labeled probes were generated by PCR using a standard kit and protocol (Roche). The primers were 5'-TTACCGGCTGAGTGTCCATAACAGG-3' and 5'-CATTCTGACGTCCCTTCCGAGTTCC-3', and the annealing temperature was 66°C. PCR amplicons from the PolyA tail length assay were run on a 1.5% agarose gel, transferred and cross-linked to a positively charged nylon membrane by UV light. After probe hybridization, the membrane was washed and blocked with a standard kit (Roche, Indianapolis, IN), and 1:10000 Anti-Digoxigenin-AP conjugate was applied. The membrane was washed again and developed with CDP-Star (Roche, Indianapolis, IN). The membrane was exposed for 0.5–15 min and developed.

### Statistical analysis

Ipsilateral and contralateral data sets were checked for normality using Shapiro-Wilkes and Liliefors tests, with p < 0.05 accepted as a non-normal distribution. Comparisons of two groups with normal distributions were made using Student's t-test. Comparisons of a non-normally distributed group with any other group were made using the Mann-Whitney U test. Comparisons of more than two groups, all of which were normally distributed, were made using 1-way ANOVA with Tukey post-hoc tests for pairwise comparisons. Two-way ANOVA with Tukey post-hoc tests was used for behavioral and electrophysiological data where a second variable (i.e. stimulus strength or time point) was analyzed simultaneously with laterality (i.e. ipsilateral vs. contralateral).

## Competing interests

The authors declare that they have no competing interests.

## Authors' contributions

DKT, IN, and IS conceived of, designed, and coordinated the study. DKT, IN, and IS interpreted the results. DKT drafted the manuscript and performed: immunohistochemistry, confocal microscopy, image analysis, cell culture, real-time PCR, and polyA tail assay experiments as well as statistical analyses. AL performed real-time PCR and 3' RACE experiments as well as statistical analyses. YM and IS prepared SNE animals. YM performed electrophysiology experiments and statistical analyses. TO performed behavioral testing. EM performed immunohistochemistry. SR performed real-time PCR and statistical analyses. IS and IN edited and helped to draft the manuscript. All authors read and approved the final manuscript.
